# Harnessing extracellular vesicles for stabilized and functional IL-10 delivery in macrophage immunomodulation

**DOI:** 10.1016/j.vesic.2025.100102

**Published:** 2025-12-12

**Authors:** Najla A. Saleh, Matthew A. Gagea, Xheneta Vitija, Sadhana Kilangodi, Ahmed A. Zarea, Tomas Janovic, Jens C. Schmidt, Cheri X. Deng, Masamitsu Kanada

**Affiliations:** a Institute for Quantitative Health Science and Engineering (IQ), Michigan State University, East Lansing, MI, USA; b Lyman Briggs College, Michigan State University, East Lansing, MI, USA; c College of Engineering, Michigan State University, East Lansing, MI, USA; d College of Natural Science, Michigan State University, East Lansing, MI, USA; e Cell and Molecular Biology Program, Michigan State University, East Lansing, MI, USA; f Department of Obstetrics and Gynecology, and Reproductive Biology, Michigan State University, East Lansing, MI, USA; g College of Human Medicine, Michigan State University, East Lansing, MI, USA; h Department of Biomedical Engineering, University of Michigan, Ann Arbor, MI, USA; i Department of Mechanical Engineering, University of Michigan, Ann Arbor, MI, USA; j Department of Pharmacology & Toxicology, Michigan State University, East Lansing, MI, USA

**Keywords:** Extracellular vesicles, Interleukin-10, Cytokine stability, Immunomodulation, Exosome purification

## Abstract

Extracellular vesicles (EVs) are gaining recognition as promising therapeutic carriers for immune modulation. We investigated the potential of EVs derived from HEK293FT cells to stabilize and deliver interleukin-10 (IL-10), a key anti-inflammatory cytokine. Using minicircle (MC) DNA vectors, we achieved IL-10 overexpression and efficient incorporation into filter-isolated small EVs (F-sEVs), resulting in superior stability compared to free recombinant IL-10. Detailed biophysical and functional analyses revealed that IL-10^+^ F-sEVs contain both monomeric and oligomeric IL-10 on their external surface and encapsulated within vesicles. Size-based fractionation of IL-10^+^ large EVs (lEVs), small EVs (UC-sEVs), and non-vesicular extracellular particles (NVEPs) revealed IL-10 presence across all fractions, predominantly in monomeric form. Anion exchange chromatography successfully enriched IL-10^+^ exosomes that efficiently associated with both IL-10 monomers and oligomers. IL-10^+^ F-sEVs suppressed inflammatory cytokine expression in pro-inflammatory macrophages (two-to 14-fold more effectively than naïve F-sEVs) without inducing anti-inflammatory repolarization. However, detailed analysis of IL-10-loaded EV subpopulations revealed that anti-inflammatory activity was distributed across multiple fractions. Moreover, naïve F-sEVs derived from non-transfected cells also exhibited anti-inflammatory effects, suggesting that endogenous EV cargo contributes to their immunomodulatory activity and complicates attribution of effects specifically to IL-10. These findings highlight the therapeutic potential of EVs while emphasizing the need to disentangle contributions of engineered cytokines from endogenous vesicular components.

## 1. Introduction

Extracellular vesicles (EVs) are emerging as central players in intercellular communication, with significant potential for diagnostics and therapeutics across various diseases, particularly inflammatory and immune-mediated disorders.^1^ EVs are categorized based on their biogenesis, size, and composition, with exosomes and ectosomes being the most well-studied subtypes. Exosomes originate from the endosomal pathway, whereas ectosomes bud directly from the plasma membrane, reflecting their diverse formation mechanisms.^2^ They carry a wide array of cargo, such as proteins, lipids, and nucleic acids, which can modulate recipient cell function upon uptake.^3^ As natural carriers of biomolecules, EVs offer unique therapeutic advantages such as high biocompatibility, stability in circulation, and an intrinsic ability to target specific cells or tissues.^4^ Recent studies have also demonstrated that EVs can be engineered to display specific protein receptors as molecular decoys, effectively sequestering pro-inflammatory cytokines—a promising approach for treating conditions characterized by immune dysregulation.^5^ In addition, EVs naturally transport various cytokines and other signaling molecules, offering a biological solution that may overcome limitations of traditional cytokine-based immunotherapies.^6^

Among various cytokines, interleukin-10 (IL-10) has garnered attention for its potent anti-inflammatory effects, making it a promising candidate for immune modulation. IL-10 is crucial in maintaining immune homeostasis, primarily by suppressing pro-inflammatory cytokine production and regulating immune cell function.^7^ However, the clinical application of IL-10-based therapies has been significantly limited by the inherent instability and rapid clearance of recombinant IL-10 protein in vivo. These limitations necessitate high therapeutic doses to achieve clinical efficacy, consequently increasing the risk of adverse effects.^8^ Delivering IL-10 protein by EVs offers a potential solution to these challenges, as the vesicular encapsulation can protect IL-10 from degradation and enhance its bioavailability.^9^

Macrophages play a central role in regulating immune responses and represent a key target for IL-10-based therapies due to their high expression of IL-10 receptors and tissue localization.^10^ These plastic cells can polarize into a pro-inflammatory (M1) or anti-inflammatory (M2) phenotype in response to environmental cues, with M1 macrophages being the primary mediators of inflammatory responses.^11^ Chronic inflammation is often associated with an imbalance favoring M1 macrophage activity, which contributes to the pathology of autoimmune and inflammatory diseases.^12^ Recent studies have demonstrated that EVs from certain sources can repolarize macrophages toward an anti-inflammatory, tissue-repair phenotype, offering a promising therapeutic strategy for treating inflammatory conditions with a predominant macrophagic component.^13–15^

We used minicircle (MC) DNA vectors to overexpress IL-10 protein and effectively incorporate it into EVs derived from HEK293FT cells. MCs encoding IL-10 allow better transfection efficiencies and prolonged transgene expression with lower cytotoxicity than plasmids.^16^ Recent studies have demonstrated that plasmid-based overexpression of cytokines in cells is an effective strategy for EV-mediated immunomodulation. However, the mechanisms of cytokine packaging into EVs and their functional delivery to recipient immune cells remain largely unclear.^9,17,18^ Our study evaluated the biophysical and biochemical properties of IL-10^+^ EVs, including their stability, interaction with pro-inflammatory macrophages, and capacity to modulate inflammatory cytokine expression. We observed that overexpressed IL-10 protein was not only incorporated as internal cargo but also present in a highly oligomerized form on the vesicle surface. EV-associated IL-10 protein exhibited enhanced stability and bioactivity, effectively suppressing pro-inflammatory markers in macrophages. Interestingly, even naïve EVs from non-transfected cells demonstrated significant anti-inflammatory effects, highlighting the complexity of extracellular signaling. These findings underscore how purification methods and the intrinsic heterogeneity of EV populations, including diverse bioactive cargo and co-isolated non-vesicular extracellular particles (NVEPs), can influence EV-mediated therapeutic delivery.

## 2. Methods

### 2.1. Plasmid construct and cloning

All plasmids were constructed using standard PCR cloning methods and verified by sequencing (Genewiz, South Plainfield, NJ). For minicircle (MC) production, *E. coli* strain, ZYCY10P3S2T, and the empty parental plasmid, pMC.BESPX-MCS2, were purchased from System Biosciences (Palo Alto, CA). The cytomegalovirus (CMV) promoter was amplified via PCR and subcloned into pMC.BESPX-MCS2. The DNA template of human IL-10 was synthesized as gBlock (Integrated DNA Technologies) and combined with mScarlet PCR fragments^19^ (Addgene plasmid #85042, gift from Dorus Gadella) to generate MC-IL-10-RFP by overlap extension PCR. MC was produced following the established protocol.^20^ Briefly, ZYCY10P3S2T was transformed with the parental plasmid and pre-cultured overnight in LB medium containing kanamycin (50 μg/mL). The culture was then expanded overnight in TB medium with kanamycin (50 μg/mL) overnight. MC production was induced by adding equal volumes of LB containing 0.01 % L-arabinose and 0.04 mol/L NaOH, followed by 6 h incubation at 32 °C. For the EV reporter, pKT2/CAGXSP/PalmReNL was previously developed^21^ (Addgene plasmid #182970). Human CD63-GFP (Addgene plasmid #62964, gift from Paul Luzio) was subcloned into the pKT2/CAGXSP vector using recombination cloning (In-Fusion HD Cloning Kit, Clontech), as described before (Addgene plasmid #231709).^22^ The DNA templates of human CD9 and CD81 were synthesized as gBlocks fused with GFP by PCR and cloned into pcDNA 3.1 (+) vector (V79020, Invitrogen).

### 2.2. Cell culture and lentiviral transduction

HEK293FT cells (R700-07, Invitrogen) and RAW 264.7 cells (ATCC) were cultured in Dulbecco’s Modified Eagle Medium (DMEM; Gibco, 11995065) supplemented with 10 % (vol/vol) fetal bovine serum (FBS; Cytiva, SH30071.03) and 1 % penicillin/streptomycin (Gibco, 15070063). THP-1 cells (kindly provided by Contag Lab) and human peripheral blood mononuclear cells (PBMCs; IQ Biosciences) were cultured in RPMI 1640 Medium (Gibco, 11875093) supplemented with 10 % FBS (vol/vol) and 1 % penicillin/streptomycin. Primary monocytes were isolated from the PBMCs by magnetic separation using the MojoSort^™^ Human Pan Monocyte Isolation Kit (BioLegend, 480059) following the manufacturer’s instruction. All cells were incubated at 37 °C in a 5 % CO_2_ atmosphere. NF-κB reporter THP-1 cells were generated using lentiviral vectors. These vectors were produced in HEK293FT cells transfected with three plasmids: pHAGE NFkB-TA-LUC-UBC-GFP-W (Addgene plasmid #49343, gift from Darrell Kotton), psPAX2 (Addgene plasmid #12260, gift from Didier Trono), and pMD2. G (Addgene plasmid #12259, gift from Didier Trono) according to the protocol described by Addgene (https://www.addgene.org/protocols/lentivirus-production/) with slight modifications. Plasmid transfection was performed using TransIT-2020 DNA transfection reagent (Mirus Bio, MIR 5400) instead of 25 kDa linear polyethyleneimine (PEI).

### 2.3. Macrophage differentiation

Macrophages were differentiated from THP-1 cells and PBMC-derived monocytes. THP-1 cells were seeded at a density of 10 × 10^6^ cells/mL and treated with 50 ng/mL phorbol 12-myristate 13-acetate (PMA; Sigma-Aldrich, P8139) for 72 h to induce macrophage-like differentiation. Following PMA treatment, cells were washed twice with phosphate-buffered saline (PBS) to remove residual PMA and incubated in fresh RPMI-1640 medium without PMA for an additional 72 h to allow stabilization. The primary human monocytes were differentiated into macrophages via Macrophage Colony-Stimulating Factor human (M-CSF, 40 ng/mL; Sigma-Aldrich, M6518). To polarize the differentiated cells into an M1-like pro-inflammatory phenotype, cells were further stimulated with 100 ng/mL lipopolysaccharide (LPS; Sigma-Aldrich, LPS25) overnight. Following polarization, M1-like macrophage differentiation was confirmed using qPCR to measure the expression of pro-inflammatory markers, including TNF-α, IL-6, and IL-1β.

### 2.4. Transfection

HEK293FT cells were transfected with MC-IL10 (with or without an RFP-tag), PalmReNL, CD9-GFP, CD63-GFP, or CD81-GFP using the TransIT-2020 transfection reagent following the manufacturer’s protocol with modifications. Briefly, on the same day, 2.5 μg/well of DNA vectors were diluted in 250 μL/well of Opti-MEM (Gibco, 31985062) and gently mixed. Subsequently, 7.5 μL/well of TransIT-2020 reagent was added to the DNA solution, mixed by gentle pipetting, and incubated for 15–20 min at room temperature to allow complex formation. The transfection complex was then added dropwise to each well containing trypsinized HEK293FT cells (2 × 10^6^ cells/well; 6-well plate) in 2.25 mL of complete medium. Following transfection, cells were incubated for 24–48 h before being split into five 100 mm tissue culture dishes (10 mL each) to reduce confluency and increase total culture volume for subsequent EV isolation.

### 2.5. EV isolation

EVs were isolated from 50 mL of cell supernatant derived from approximately 4 × 10^7^ cells, using two different methodologies. For both methods, EV-depleted FBS was prepared by ultracentrifugation at 100,000×*g*, 4 °C for 18 h.^23^ After transfected and non-transfected (control group) HEK293FT cells reached around 50 % confluence, the media were changed to EV-depleted media or serum-free media (without FBS), and the cells were cultured for an additional 72 h to collect the conditioned media. *First isolation method - Filter-isolated small EVs (F-sEVs):* sEVs-enriched fractions were isolated as previously described^21^ with modifications as described. The conditioned media were centrifuged at 600×*g* for 5 min to remove cells and large debris. Subsequently, to remove large vesicles, the supernatants were filtered through 0.2 μm PES membrane filters (Nalgene, 725–2520). Finally, sEV fractions were collected by a size-based EV isolation method using 50-nm porous membranes (Whatman, WHA110603) with holders (EMD Millipore, SX0002500) by applying a vacuum pressure.^24^ The concentrated F-sEV fractions were washed with 5 mL PBS and collected from the membranes. *Second isolation method* – *Ultracentrifugation-based fractionation (UC):* large EV (lEV)-, UC-sEV-, and non-vesicular extracellular particle (NVEP)-enriched fractions were isolated as previously described^25^ with modifications. The conditioned media were centrifuged at 2000×*g* for 20 min to remove cells, debris, and apoptotic bodies. The remaining supernatant was centrifuged at 10,000×*g* for 30 min to collect the pellet containing lEVs. The pellet of lEVs was washed with PBS at 10,000×*g* for 30 min. The supernatant from the first 10, 000×*g* centrifugation was filtered through 0.2 μm PES membrane filter and ultracentrifuged at 100,000×*g* for 50 min to collect the pellet containing UC-sEVs. The pellet of UC-sEVs was washed with PBS at 100, 000×*g* for 50 min. The supernatant from the first 100,000×*g* centrifugation was concentrated from 50 mL to 2.5 mL using Amicon^®^ Ultra Centrifugal Filters (10 kDa MWCO, Millipore, UFC8010) at 10,000×*g* for 5 min. The concentrated supernatant was washed with PBS to collect the NVEP fraction. To ensure the stability of EVs/NVEPs under freezing conditions, a 10 % freezing solution containing 250 mM trehalose, 250 mM HEPES, and 2 % bovine serum albumin (BSA) was used as a cryoprotective agent.^26^ The isolated fractions were aliquoted into 200 μL portions and stored at −80 °C until analysis.

### 2.6. EV purification by anion-exchange column chromatography

The F-sEVs isolated from the initial methodology underwent additional purification using anion-exchange column chromatography to analyze exosome-enriched fractions. Using a HiTrap DEAE Sepharose Fast Flow column (Cytiva, 17505501), 1 mL of pre-isolated F-sEVs (from 20 mL conditioned medium) was processed as previously demonstrated with modifications.^27^ The anion-exchange column was first equilibrated with an equilibration buffer (10 mM Tris-HCl, pH 7.4, containing 30 mM NaCl). The F-sEV-containing PBS solution was diluted 5-fold with 10 mM Tris-HCl (pH 7.4) to adjust the NaCl concentration before column loading. After washing with equilibration buffer, EVs bound to DEAE-sepharose were eluted using a linear NaCl gradient (30–500 mM) and collected in 500 μL fractions, 20 fractions in total (10 mL). Electrical conductivity and UV absorbance measurements for each fraction were obtained using an ÄKTA pure HPLC system (GE Life Sciences). Based on UV absorbance profiles, the fractions were pooled into three groups: Protein-high (2 mL), Protein-low (8 mL), and flow-through (10 mL). Each pooled fraction was concentrated to 1 mL using Amicon^®^ Ultra Centrifugal Filters (100 kDa MWCO, Millipore, UFC8100).

### 2.7. Nanoparticle tracking analysis (NTA)

The size distribution and concentration of EVs/NVEPs were determined using NTA (ZetaView, Particle Metrix). Samples were diluted in PBS to achieve optimal particle concentration (1 × 10^9^−1 × 10^10^ particles/mL) before analysis. Automated measurements were taken with outlier control to select optimal video quality. Particle size distribution (diameter in nm) and concentration (particles/mL) were calculated based on Brownian motion and light scattering principles.

### 2.8. Protein quantification

Protein quantification was performed using the Micro Bicinchoninic Acid (BCA) Assay (Thermo Fisher Scientific, 23235), according to the manufacturer’s protocol. Briefly, 1 μL of EVs/NVEPs was diluted in 99 μL PBS and incubated with the BCA reagent working reagent at 37 °C for 2 h. Absorbance was measured at 562 nm. A standard calibration curve was generated using bovine serum albumin (BSA) standards ranging from 0.5 to 40 μg/mL, and protein concentrations were determined by linear regression based on the standard curve.

### 2.9. Western blotting

The isolated EVs/NVEPs [in equal amounts of protein (30 μg) or volume (15 μL)] were lysed with 4X sample buffer (Bio-Rad, 1610747) with (for detecting TSG101 and IL-10) or without β-mercaptoethanol (for detecting CD81, CD63, and CD9), ran on a 4–20 % Mini-PROTEAN TGX Stain Free gel (Bio-Rad, 4568096), and transferred to PVDF membranes (Millipore, IPFL00010). The parental cells were also lysed to verify IL-10 and GAPDH. Membranes were blocked with PBS containing 5 % skim milk and 0.05 % Tween20 (v/v) for 30 min at room temperature; incubated with primary antibodies overnight at 4 °C at dilutions recommended by the suppliers as follows: anti-IL-10 (1:5000; Proteintech, 60269-1-lg), anti-GAPDH (1:10,000; Santa Cruz, sc-365062), anti-TSG101 (1:1000; Proteintech, 14497), anti-CD81 (1:3000; Proteintech, 66866), anti-CD9 (1:1000; Proteintech, 60232), anti-CD63 (1:1000; Ts63, Thermo Fisher, 10628D), anti-Calnexin (1:1000; Cell Signaling, 2679T), Membranes were washed 3 times with PBS containing 0.05 % Tween20 (v/v), incubated with HRP-conjugated anti-mouse (1:10,000; Cell Signaling, 7076) or anti-rabbit (1:10,000; Cell Signaling, 7074) antibodies for 1 h at room temperature, and washed again to remove unbound antibodies. Membranes were visualized with ECL Select Western Blotting Detection Reagent (GE Healthcare, RPN2235) on ChemiDoc MP Imaging System (Bio-Rad).

### 2.10. Enzyme-linked immunosorbent assay (ELISA)

Human pro-inflammatory cytokines—TNF-α, IL-1β, and IL-6—were quantified in the cell supernatant using commercially available ELISA kits (BioLegend, San Diego, CA, USA) according to the manufacturer’s instructions. TNF-α levels were measured using the Human TNF-α ELISA MAX^™^ Standard Set (Cat. No. 430201). IL-1β concentrations were determined using the Human IL-1β ELISA MAX^™^ Deluxe Set (Cat. No. 437004). IL-6 levels were assessed using the Human IL-6 LEGEND MAX^™^ Standard Set (Cat. No. 430501).

### 2.11. Fluorescence microscopy

Fluorescence deconvolution images were taken using the DeltaVision Microscope system (GE Healthcare Life Sciences). Cells (5 × 10^4^) were observed using a glass bottom μ-dish 35 mm (ibidi, 81156). To analyze F-sEV uptake, 4 × 10^9^ IL-10-RFP^+^ sEVs were incubated with the cells for 17 h. The cells were stained with 10 μg/mL Hoechst 33342 (Life-Technologies, H3570) before microscopy was performed. The fluorescence filter set for TRITC and DAPI and the 60× oil immersion objective, 1.42 NA, were used to acquire images and process z-stacks (Optical section space: 0.2 μm, Number of optical sections: 30) for deconvolution. Maximum intensity projection images of the z-stack were created using ImageJ software (The National Institutes of Health, Bethesda, MD, USA). To quantify the CD63/CD9/CD81-GFP-positive and IL-10-RFP-positive sEVs, a droplet of the isolated sEVs was applied onto hydrophobic PTFE printed slides (Electron Microscopy Sciences, 63429-04), as previously described.^28^ After 30 min of incubation at 4 °C, the slides were gently rinsed twice with PBS, and imaging was subsequently performed. Image processing and quantification were carried out using ImageJ/Fiji software^29^ with the EVAnalyzer plugin ^30^. The ‘EVColoc’ function was utilized to determine the number of RFP and GFP-positive sEVs. A statistical analysis was performed on the data produced by EVAnalyzer using an IPython script with thresholds set using the Median ±1.5*IQR metric.

### 2.12. Transmission electron microscopy

The isolated EVs/NVEPs were fixed in 1 % paraformaldehyde to assess the sample morphology. A formvar-coated gold grid was pre-conditioned in a humidified chamber for 24 h and placed onto a 50 μL droplet of the EV solution and incubated, covered, for 20 min. Following incubation, grids were washed and blocked by sequential placement—face down—on droplets of the following solutions: PBS (2 ×, 3 min), PBS/50 mM Glycine (4 ×, 3 min), PBS/5 % BSA (1 ×, 10 min). EVs were labeled with an anti-IL-10 antibody (1:200; Proteintech, 60269-1-lg) diluted in 5 % BSA/PBS for 1 h, followed by six washes in PBS/0.5 % BSA. Grids were then incubated with a 1:50 dilution of anti-mouse IgG-gold (Sigma-Aldrich, G6652) in 5 % BSA/PBS (20 min) and washed in PBS (6 ×) and water (6 ×). For negative staining, 1 % uranyl acetate was applied, and the excess stain was carefully wicked off using filter paper. Grids were air-dried and imaged using a JEOL 1400 Flash Transmission Electron Microscope equipped with an integrated Mata-taki Flash sCMOS bottom-mounted camera. The 1400 Flash was operated at 100 kV.

### 2.13. Proteinase K protection assay

To determine the localization of IL-10 within EVs/NVEPs, aliquots containing 2 × 10^8^ particles were incubated with proteinase K (final concentration 10 μg/mL; Qiagen, 19134) in PBS to a final volume of 50 μL, as previously described.^31^ The samples were kept on ice for 20 min, followed by treatment with or without 1 % Triton X-100 on ice for 10 min. The reactions were halted by adding Halt Protease Inhibitor Cocktail (to a final concentration of 1 ×; Thermo Fisher, 87786). Samples were subsequently prepared for SDS-PAGE and analyzed by Western blotting.

### 2.14. Binding of human IL-10 recombinant protein (hIL-10 RP) to sEVs

Following established protocols,^32^ F-sEVs (2 × 10^9^ particles) or PBS (control group) were incubated with 1 μg/mL of hIL-10 RP (Cell Signaling, 35979) for 2 h at 37 °C under constant agitation. Unbound hIL-10 RP was removed by concentrating the F-sEVs with Amicon^®^ Ultra Centrifugal Filters, 100 kDa MWCO (Millipore, UFC8100). The concentrated samples were then prepared for SDS-PAGE and analyzed by Western blotting.

### 2.15. Treatment of sEVs with heparinase II

To investigate the IL-10 protein binding to heparan sulfate, F-sEVs (2 × 10^9^ particles) were incubated with 40 U/mL of Heparinase II (Sigma-Aldrich, H6512) in a final volume of 200 μL for 2 h at 37 °C under agitation, as described in previous studies.^33^ Control F-sEVs were incubated with PBS alone. The reactions were terminated by adding Halt Protease Inhibitor Cocktail to a final concentration of 1 ×. Samples were then processed for SDS-PAGE and analyzed by Western blotting.

### 2.16. Inhibition of glycosaminoglycan (GAG) synthesis

To inhibit glycosaminoglycan (GAG) synthesis, HEK293 cells transfected with IL-10 were treated with 2.5 mM of the pharmacological inhibitor p-Nitrophenyl-β-D-xylopyranoside (pNP-Xyl; Sigma-Aldrich, 487870) for 72 h, as previously reported.^34^ Following treatment, cell supernatants were collected for F-sEVs isolation and analyzed by Western blotting.

### 2.17. Bioluminescence assays

The uptake of IL-10^+^ PalmReNL-sEVs and the NF-κB activity in M1-like macrophages were analyzed by bioluminescence measurement after incubating the cells with EVs/NVEPs. Wild-type THP-1 cells or NF-κB-reporter THP-1 cells were plated in 96-well black clear-bottom cell culture plates at a concentration of 50,000 cells/well, with or without polarization to M1-like macrophages via 100 ng/mL LPS stimulation. The culture medium was replaced with EV-depleted medium or serum-free medium, and IL-10^+^ PalmReNL-sEVs (8 × 10^8^ particles) or IL-10^+^ EVs/NVEPs (20 μL or 2–20 μg protein) were added at the indicated concentrations, with or without pre-incubation with anti-IL-10 receptor alpha antibodies (1 μg/mL; R&D Systems, MAB274). After the incubation period, the cells were washed twice with PBS, and the uptake of PalmReNL-sEVs and NF-κB activity were analyzed by measuring bioluminescence signals. Furimazine (25 μM; Promega, N1110) was added for PalmReNL detection, and D-luciferin (300 μg/mL; Promega, E160A) was added for the NF-κB reporter. Assays were performed using a Spark Multimode Microplate Reader (Tecan).

### 2.18. Quantitative real-time PCR (qPCR)

Total RNA was extracted from isolated cells and EVs/NVEPs using the RNeasy Mini Kit (Qiagen, 74106) and Exosomal RNA Isolation Kit (Norgen Biotek, 58040), respectively, according to the manufacturer’s protocols. RNA concentration and purity were assessed using a Nano-Drop spectrophotometer (Thermo Fisher Scientific). cDNA synthesis was performed with 25 ng of RNA using the SuperScrip III Reverse Transcriptase (Invitrogen, 56575) in a 20 μL reaction volume following the manufacturer’s instructions. qPCR was conducted using the SYBR Select Master Mix for CFX (Applied Biosystems, 447942) on a CFX96 RealTime System (Bio-Rad). Each reaction was performed in triplicate in a total volume of 20 μL, containing 1 μL of diluted cDNA template. Primers were designed to span exon-exon junctions to prevent amplification of genomic DNA (Supplementary Table 1). Relative expression levels were calculated using the ΔΔCt method, with normalization to the house-keeping gene GAPDH or CD68. Data analysis was conducted using BioRad CFX Maestro software, and fold changes were calculated relative to the control group. All experiments were conducted in biological triplicates, and data were presented as mean ± standard error.

### 2.19. Statistical analysis

All statistical analyses were performed using GraphPad Prism version 10 (GraphPad Software, Inc.). Data are presented as mean ± standard error. For all statistical tests, a p-value <0.05 was considered statistically significant. Comparisons between the two groups were conducted using an unpaired two-tailed Student’s t-test. For experiments involving comparisons among more than two groups, a one-way analysis of variance (ANOVA) was performed, followed by Tukey’s post-hoc test for multiple comparisons, where appropriate. A two-way ANOVA was applied to assess the effect of two independent variables on a dependent variable. Interaction effects were evaluated, and post-hoc comparisons were performed using Šídák’s correction for multiple testing if a significant interaction was detected. All experiments were conducted with at least three biological replicates, and statistical analysis was carried out on data from independent experiments. Graphical representation of the data was generated using GraphPad Prism, with detailed descriptions provided in figure legends.

## 3. Results

### 3.1. IL-10 protein exists as stabilized monomers and oligomers both on the surface and within filter-isolated small EVs (F-sEVs)

**Fig. 1.**
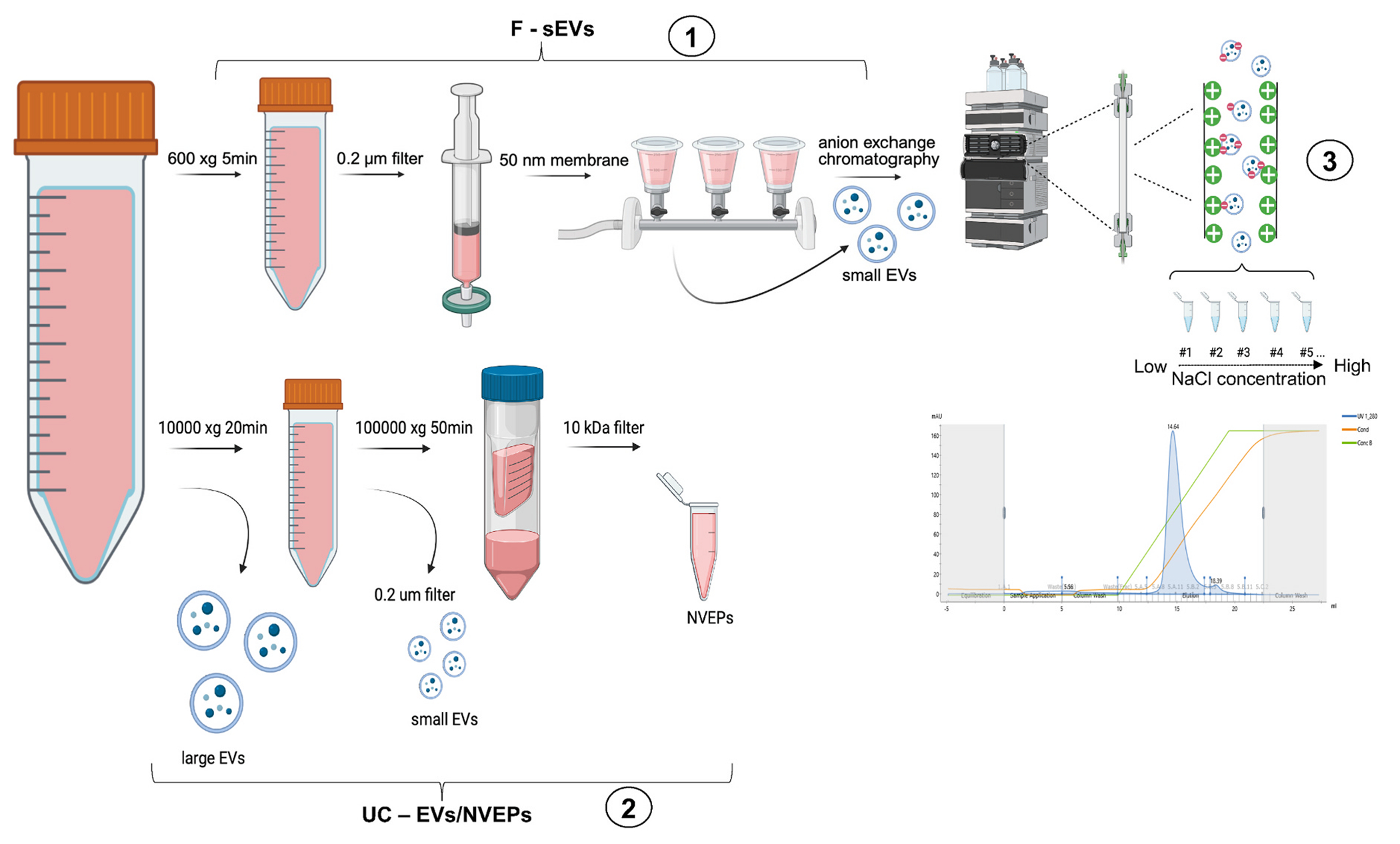
Workflow for EV isolation and purification strategies used in IL-10^+^ vesicle analysis. Conditioned media from IL-10-transfected and non-transfected (naïve) HEK293FT cells underwent three complementary isolation procedures: (**1**) Size-based filtration enriched small extracellular vesicles (F-sEVs) via 0.2 μm filtration followed by vacuum filtration through 50-nm porous membranes; (**2**) Differential ultracentrifugation (UC) fractionated extracellular particles into large EVs (lEVs, 10K pellet), UC-sEVs (100K pellet after 0.2 μm filtration), and non-vesicular extracellular particles (NVEPs, 10 kDa-concentrated supernatant); (**3**) Anion exchange chromatography further purified F-sEVs based on surface charge, yielding protein-high, protein-low, and flow-through fractions. Created with BioRender.com.

To achieve overexpression of human IL-10, we constructed a minicircle DNA vector encoding IL-10 (MC-IL-10) with or without a red fluorescent protein (RFP) tag (Supplementary Fig. S1a). These constructs were transfected into human embryonic kidney (HEK293FT) cells, a commonly used EV producer cell line selected for its high EV yield, suitability for biomanufacturing (e.g., lentivirus production), and reduced variability compared to primary cells with donor-to-donor heterogeneity. Punctate IL-10-RFP signals were observed in the cytoplasm and near the plasma membrane, indicating that IL-10 was associated with discrete subcellular structures (Supplementary Fig. S1b). Successful overexpression of IL-10 was confirmed at both protein and mRNA levels (Supplementary Fig. S1c). To evaluate the expression kinetics of MC compared to their parental plasmid (PP) counterparts, we transfected HEK293FT cells with equivalent DNA masses of PP-IL-10-RFP or MC-IL-10-RFP and monitored fluorescence over time. MC vectors produced stronger and more sustained RFP expression from day 2 to day 6 post-transfection (Supplementary Fig. S1d). Quantitative fluorescence measurements confirmed significantly higher and more prolonged expression levels from MC-IL-10-RFP constructs compared to PP-IL-10-RFP (Supplementary Fig. S1e). We then isolated EVs from the culture conditioned medium of MC-IL-10-transfected HEK293FT cells. To evaluate how isolation methods influence IL-10 loading and vesicle function, we compared three complementary EV purification strategies: (1) size-based filtration to enrich F-sEVs; (2) differential ultracentrifugation (UC) to separate vesicle subpopulations into three distinct fractions: large EVs (lEVs), pelleted small EVs (UC-sEVs), and non-vesicular extracellular particles (NVEPs); and (3) anion exchange chromatography to further purify exosome-enriched fractions. This approach enabled direct comparison of IL-10 distribution, stability, and immunomodulatory activity across EV subsets (Fig. 1).

**Fig. 2.**
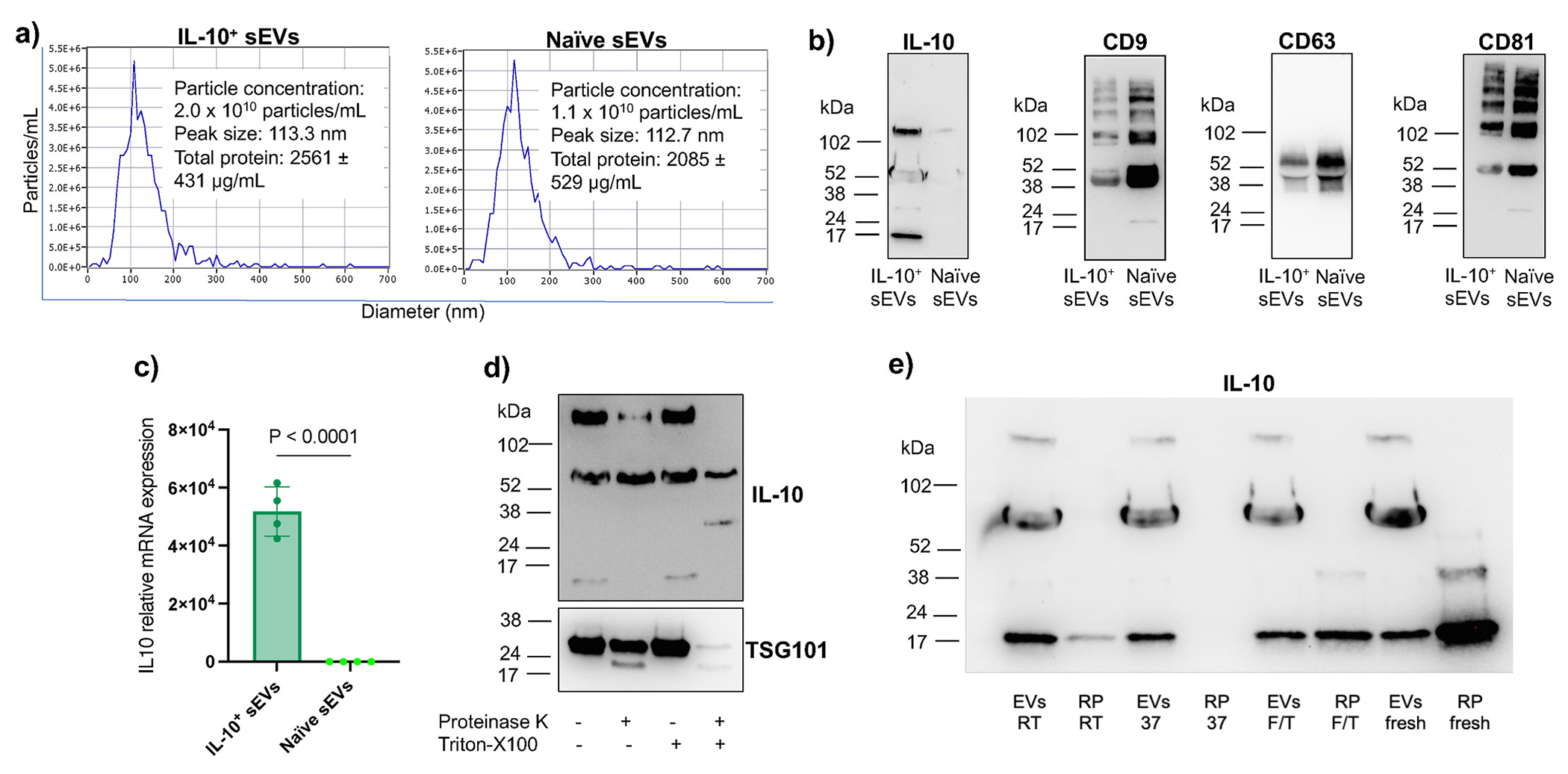
Biophysical characterization of F-sEV-associated IL-10. **a)** Size distribution histogram showing particle concentration per size interval (particles/mL). Particle concentration, peak size, and total protein concentration are indicated. **b)** Western blot analysis of IL-10 protein showing multiple bands under reducing condition, and EV markers CD9, CD63, and CD81 under non-reducing conditions. **c)** Efficient encapsulation of overexpressed IL-10 mRNA into IL-10^+^ F-sEVs was detected by quantitative PCR (qPCR) compared to the endogenous level of IL-10 mRNA in naïve sEVs. (n = 5, unpaired two-tailed Student’s t-test). d) Proteinase K protection assay was performed to determine the localization of IL-10 within F-sEVs. IL-10 was assessed in the presence or absence of proteinase K, with or without Triton X-100 treatment. TSG101, a luminal EV protein, was used as a control to confirm that intra-vesicular proteins are protected from enzymatic degradation unless the membrane is disrupted by detergent treatment. Detection was performed under reducing conditions. e) Stability comparison between F-sEV-incorporated IL-10 and recombinant human IL-10 protein (RP) under 2 h at room temperature (RT), 2 h at 37 °C (37), and 2 cycles of freezing and thawing (F/T). Fresh samples served as controls. Naïve F-sEVs were isolated from non-transfected HEK293FT cells. An equal amount (30 μg) of total protein of F-sEVs was used for Western blot analysis.

Nanoparticle tracking analysis (NTA) confirmed similar size distributions between F-sEVs isolated from IL-10-transfected and untransfected cells (113.3 nm and 112.7 nm in diameter, respectively) (Fig. 2a). The presence of IL-10 in the F-sEVs was validated by protein analysis (Fig. 2b), mRNA detection (Fig. 2c), and immunogold labeling by transmission electron microscopy (TEM) (Supplementary Fig. S2a). Interestingly, IL-10 protein associated with F-sEVs appeared as multiple bands, indicating the presence of distinct oligomeric forms (Fig. 2b). While it remains to be determined whether these oligomers represent functionally ordered structures or non-functional aggregates, we refer to them here as oligomerized IL-10 to distinguish from monomers. The isolated F-sEVs were further characterized using established markers: tetraspanins (CD9, CD63, and CD81) and TSG101 as positive EV markers, and Calnexin as a negative EV marker (Fig. 2b and Supplementary Fig. S2b). Single-EV fluorescence microscopy analysis of IL-10-RFP-positive F-sEVs revealed similar colocalization frequencies with GFP-tagged tetraspanins CD9 (2.8 %), CD63 (2.6 %), and CD81 (2.1 %) (Supplementary Fig. S2c). Since TEM detected IL-10 on F-sEV surfaces (Supplementary Fig. S2a), we performed a proteinase K protection assay to determine the membrane orientation of F-sEV-associated IL-10 (Fig. 2d). Monomeric and higher-order oligomeric IL-10 were degraded by proteinase K treatment, indicating their localization on the sEV surface. In contrast, smaller oligomeric forms were partially degraded only when combined with Triton-X100 treatment, suggesting they were at least partially protected within the vesicles. The intraluminal EV marker TSG101 remained protected from proteinase K digestion, although a slightly smaller minor band was detected under our experimental conditions. Notably, stability assays showed that both encapsulated and surface-associated IL-10 exhibited significantly greater stability compared to recombinant human IL-10 (IL-10 RP) when subjected to various stress conditions, including exposure to room temperature (2 h), physiological temperature (37 °C, 2 h), and repeated freeze-thaw cycles (Fig. 2e).

The enhanced stability of surface-associated IL-10 suggests a unique mode of sEV association, prompting further investigation into the underlying mechanisms. Since EV surface components, such as heparan sulfate proteoglycans (HSPGs), are known to mediate protein binding,^35^ we investigated whether IL-10 associates with sEVs through HSPG-mediated interactions.

### 3.2. IL-10 association with F-sEVs occurs independently of HSPGs

To explore the potential interaction between IL-10 and HSPGs on F-sEVs, we conducted several experimental approaches to investigate whether HSPG-mediated interactions influence IL-10 binding to F-sEVs. HSPGs, known to be present on the surface of cells and EVs, play a critical role in cell signaling, adhesion, and communication.^35^ HEK293FT cells were treated with 4-nitrophenyl β-D-xylopyranoside (PNP-Xyl), an HSPG biosynthesis inhibitor that disrupts proteoglycan glycosylation and reduces functional HSPG levels on the cell surface.

However, the results were inconclusive due to reduced IL-10 expression in DMSO-treated control cells (Supplementary Fig. S3a), suggesting that the treatment conditions may have affected IL-10 production or stability. This finding is consistent with reports demonstrating that DMSO concentrations as low as 0.25 % can elevate pro-inflammatory cytokine levels.^36^ To directly test whether IL-10 binding to F-sEVs depends on heparan sulfate chains, common glycosaminoglycans (GAGs) on HSPGs, we performed a heparinase assay. IL-10 levels on F-sEVs remained unchanged after treatment, indicating that its association was independent of heparan sulfate (Supplementary Fig. S3b). To further investigate IL-10 binding to F-sEVs under controlled conditions, we attempted to bind recombinant IL-10 to naïve F-sEVs based on the corona formation model,^37^ where proteins adsorb onto vesicle surfaces. However, after a 2-h incubation, no detectable IL-10 monomers were observed on the F-sEV surface (Supplementary Fig. S3c). This suggests that IL-10 either does not readily bind to naïve F-sEVs under these conditions, or that any binding that does occur is too weak or transient to be detected by our assay.

### 3.3. Size-based separation of lEVs, UC-sEVs, and NVEPs reveals IL-10 protein association with structural heterogeneity

**Fig. 3.**
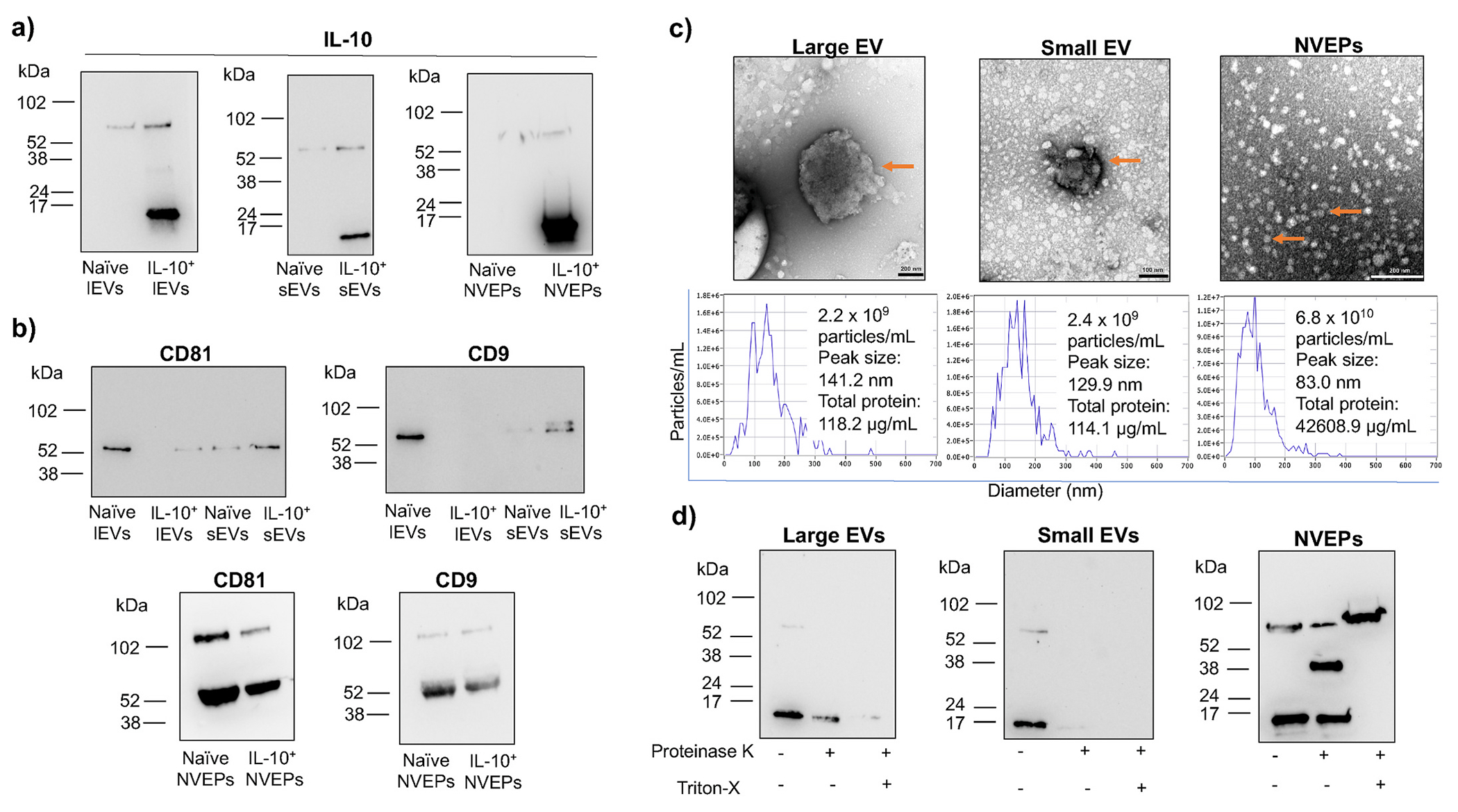
Characterization of IL-10 protein in lEVs, UC-sEVs, and NVEPs separated by differential ultracentrifugation. Western blot analysis of (**a**) IL-10 and (**b**) CD81 and CD9 protein expression. An equal volume (15 μL) of EVs/NVEPs was used for Western blot analysis. **c)** Representative TEM images of EVs and NVEPs (orange arrows) and size distribution histogram showing particle concentration per size interval (particles/mL). The particle concentration, peak size, and total protein concentration are indicated in the figure. **d)** Proteinase K protection assay characterizing IL-10 protein in IL-10^+^ EVs/NVEPs, analyzed by Western blotting (equal volumes loaded: 15 μL per lane).

The TEM images revealed small particles (<50 nm) labeled with anti-IL-10 antibodies in IL-10^+^ F-sEV samples (Supplementary Fig. S2a). To investigate how IL-10-associated EV functionality varies by particle size, we used differential UC and filter concentration to separate IL-10^+^ EVs into three fractions: lEVs, UC-sEVs, and NVEPs (Fig. 1). Protein analysis showed that IL-10 was present in all subpopulations, primarily in monomeric form (Fig. 3a). Notably, we often detected weak signals in an oligomeric form in naïve EV fractions, suggesting the existence of endogenous EV-associated IL-10. The presence of EV markers (CD9, CD81) was confirmed in IL-10^+^ EVs/NVEPs, except CD9 in lEVs (Fig. 3b). TEM and NTA analysis revealed the size and morphology, respectively, of each group (Fig. 3c). Proteinase K protection assays demonstrated differential IL-10 localization across EV populations: in lEVs, monomers were present on both internal and external surfaces, while in UC-sEVs, monomers localized primarily on the outer surface. Notably, NVEPs contained both IL-10 monomers and oligomers, with only the oligomeric forms showing partial degradation upon proteinase K treatment. Complete removal of monomeric IL-10 was observed following combined treatment with proteinase K and Triton X-100 (Fig. 3d).

### 3.4. Protein-high exosomes efficiently associate with both IL-10 monomers and oligomers

**Fig. 4.**
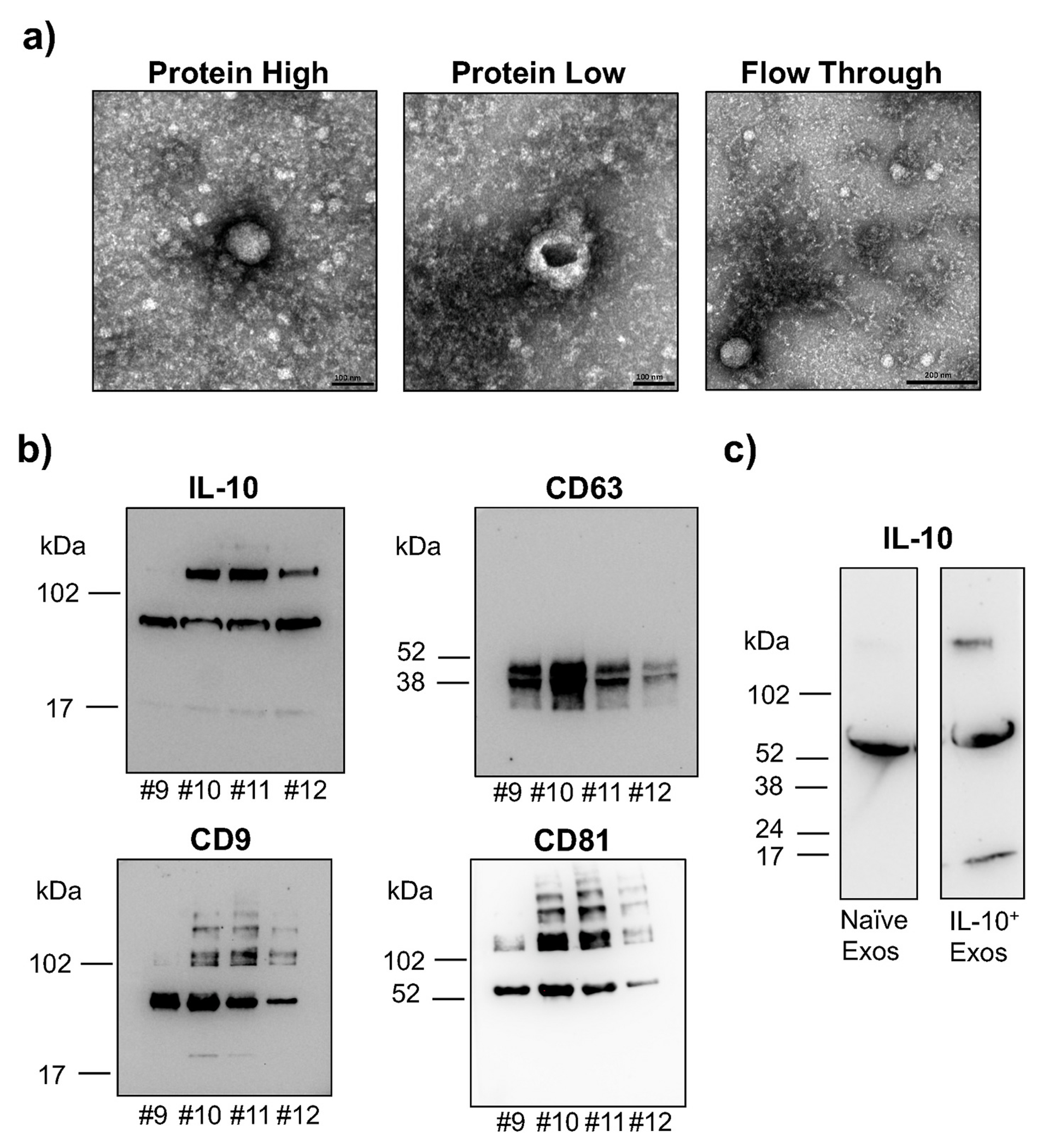
Characterization of IL-10^+^ exosomes purified using anion exchange chromatography. **a)** Representative transmission electron microscopy images of sEVs in Protein-high (left), Protein-low (middle), and Flow-through (right) fractions purified from IL-10^+^ F-sEVs. **b)** Western blot analysis of IL-10 protein (reducing conditions) and exosome markers CD9, CD63 and CD81 (non-reducing conditions) in Protein-high fractions 9–12. **c)** Western blot analysis comparing IL-10 protein expression between naïve and IL-10^+^ exosomes. Equal amounts (30 μg) of total protein were used for Western blot analysis.

Anion exchange chromatography was employed to further enhance the purity of IL-10^+^ sEVs, as demonstrated previously,^27^ enriching high-performance exosomes based on their surface charge characteristics using a linear NaCl gradient for elution (Fig. 1). Three groups of fractions were collected: Protein-high, Protein-low, and Flow-through (Fig. 4a). Following detailed characterization, four fractions within the Protein-high group exhibited significant enrichment of both IL-10 and exosome markers (CD9, CD63, and CD81) (Fig. 4b, Supplementary Fig. S4). Western blot analysis revealed that IL-10^+^ exosomes contained three distinct forms of IL-10: monomeric and two oligomeric forms. Notably, the lower molecular weight oligomeric form was also detected in naïve exosomes (Fig. 4c).

### 3.5. IL-10-loaded F-sEVs exhibit anti-inflammatory activity in pro-inflammatory macrophages

**Fig. 5.**
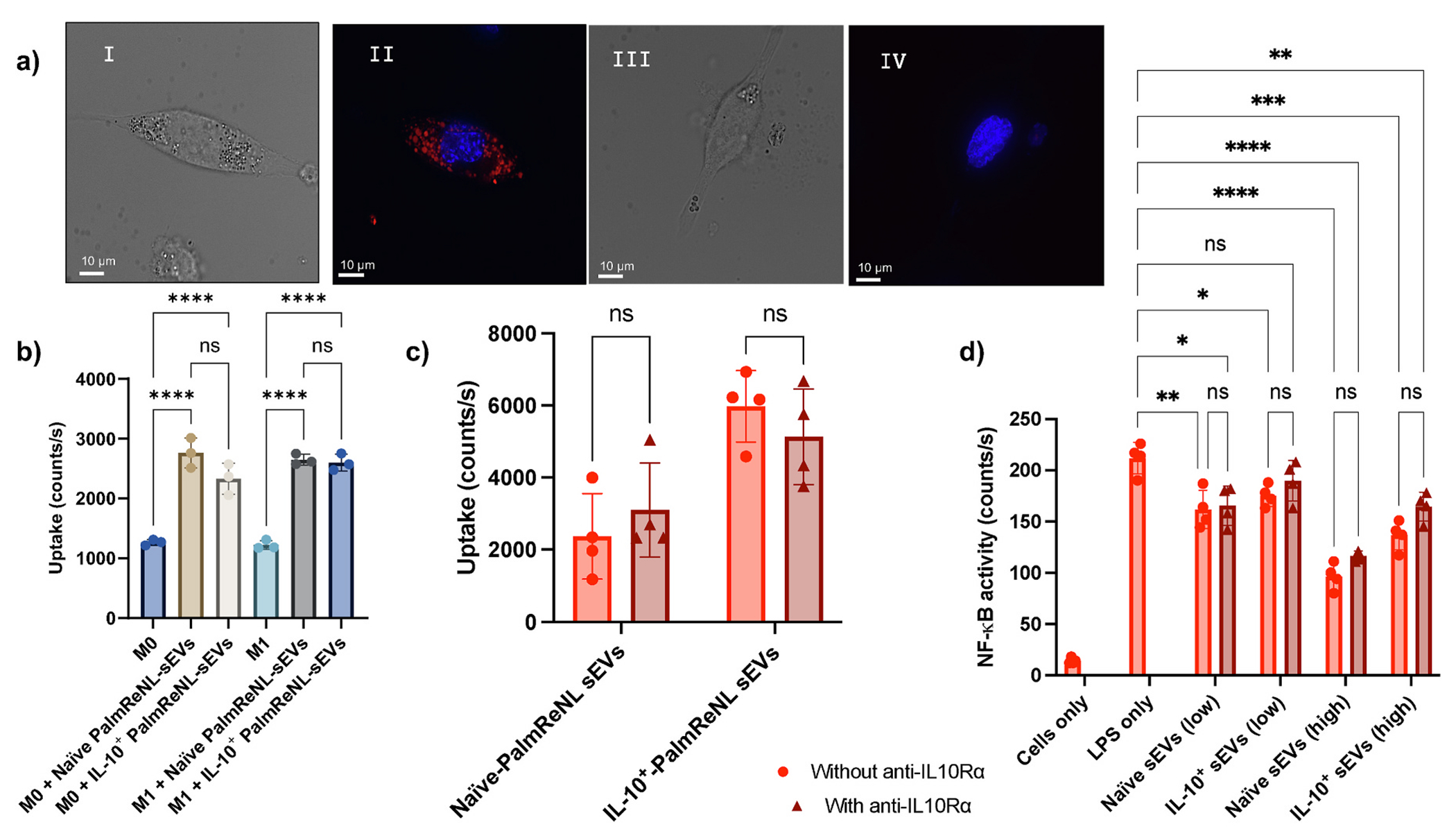
Uptake of IL-10^+^ F-sEVs by pro-inflammatory macrophages and associated suppression of NF-κB activity. **a)** Bright-field (I, III) and fluorescence (II, IV) images showing in red the accumulation of IL-10-RFP^+^ F-sEVs (I, II) and untreated controls (III and IV) in pro-inflammatory macrophages (THP-1 cells stimulated with PMA and LPS) after 17-h incubation. **b)** Uptake of naïve and IL-10^+^ PalmReNL-F-sEVs by M0 and pro-inflammatory M1 macrophages assessed by detecting bioluminescence after 2-h incubation (n = 3, one-way ANOVA). **c)** Uptake of PalmReNL-F-sEVs by M1 macrophages following pre-treatment with anti-IL-10Rα antibodies (n = 4, unpaired two-tailed Student’s t-test). d) Bioluminescence evaluation of NF-κB activity in monocytes after incubation with F-sEVs at low (2 μg protein) and high (20 μg protein) concentrations, with or without anti-IL-10Rα antibodies (n = 4, two-way ANOVA). (*) p < 0.05, (**) p < 0.01, (***) p < 0.001, and (****) p < 0.0001.

To evaluate the cellular uptake of IL-10-loaded F-sEVs by pro-inflammatory macrophages, we employed fluorescence imaging and bioluminescence quantification of reporter F-sEVs (IL-10-RFP and PalmReNL, respectively). Fluorescence microscopy indicated significant uptake of IL-10-RFP-positive F-sEVs by M1-like macrophages after 17 h, as evidenced by prominent endosomal accumulation of RFP fluorescence signals (Fig. 5a). Bioluminescence analysis using PalmReNL-labeled F-sEVs further revealed no significant difference in uptake between IL-10-carrying F-sEVs and control PalmReNL-F-sEVs (without IL-10), following 2 h of incubation with either M0 or pro-inflammatory M1-like macrophages (Fig. 5b). To investigate the role of the IL-10 receptor (IL-10R) in F-sEV uptake, macrophages were pre-treated with anti-IL-10Rα blocking antibodies. This treatment led to a modest but not statistically significant reduction in bioluminescence signals from IL-10-carrying PalmReNL-F-sEVs (Fig. 5c), suggesting that IL-10R is not the primary mechanism for IL-10^+^ F-sEV internalization. Instead, F-sEVs uptake likely occurs through receptor-independent endocytic pathways. In addition, both naïve and IL-10^+^ F-sEVs suppressed NF-κB activity regardless of anti-IL-10Rα antibody treatment (Fig. 5d), indicating shared anti-inflammatory mechanisms independent of IL-10R signaling.

**Fig. 6.**
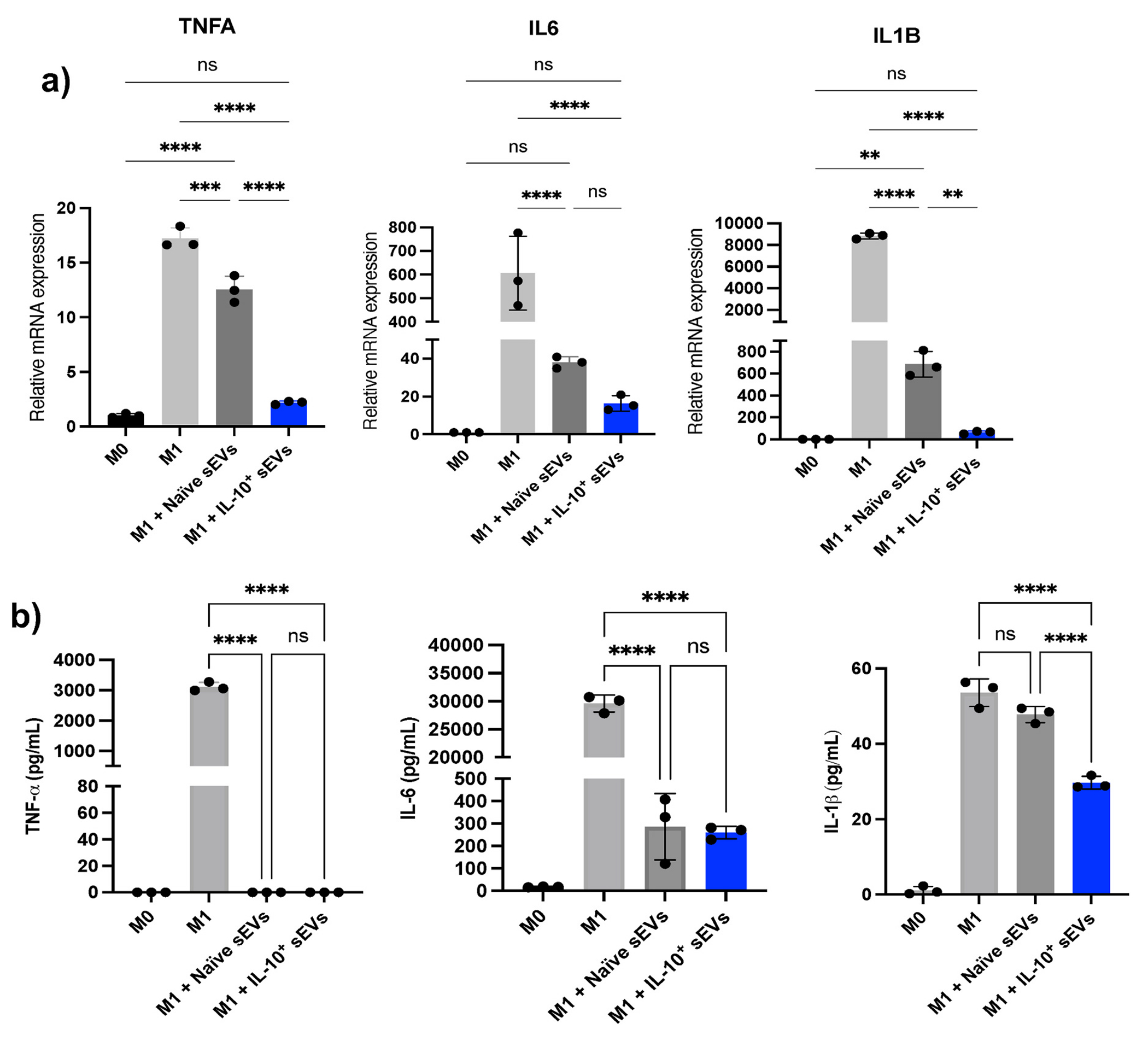
Analysis of pro-inflammatory cytokine suppression by IL-10^+^ F-sEVs at mRNA and protein levels. **a)** qPCR analysis of pro-inflammatory marker mRNA expression and **b)** ELISA quantification of pro-inflammatory cytokine concentrations in supernatants from LPS-stimulated THP-1 macrophages after 24-h incubation with 10 μg (total protein) of F-sEVs. M0: non-polarized macrophages; M1: pro-inflammatory macrophages (n = 3, one-way ANOVA). (**) p < 0.01, (***) p < 0.001, and (****) p < 0.0001.

To further examine the differential effects of naïve F-sEVs and IL-10^+^ F-sEVs, we assessed the expression of pro-inflammatory cytokines TNFA, IL6, and IL1B in LPS-stimulated (M1-like) macrophages by qPCR. IL-10^+^ F-sEV significantly reduced the expression of all three cytokines to levels comparable to those in non-polarized (M0-like) macrophages. Naïve F-sEVs also suppressed these cytokines, though to a lesser extent (Fig. 6a). Consistently, reduced secretion of these cytokines was observed at the protein level in cell supernatants (Fig. 6b). To assess whether these F-sEVs reprogram macrophages, we measured the expression of various anti- and pro-inflammatory cytokine genes following treatment with naïve or IL-10^+^ F-sEVs. IL-10^+^ F-sEVs demonstrated comparable immunomodulatory effects to recombinant IL-10 protein (IL-10-RP) in pro-inflammatory M1-like macrophages. Neither treatment increased the expression of M2-like endogenous anti-inflammatory cytokines, including IL-10, CD163, MRC1, and CD209, indicating that the anti-inflammatory effects were primarily mediated by the exogenous IL-10 protein and potentially other bioactive F-sEV cargo (Supplementary Fig. S5a). To confirm that surface-exposed IL-10 mediates the anti-inflammatory effects, IL-10^+^ F-sEVs were pre-treated with proteinase K to digest surface proteins. This treatment selectively reduced TNFA mRNA levels while leaving IL6 and IL1B expression unchanged, suggesting that surface IL-10 on F-sEVs selectively modulates TNFA expression through IL-10R-mediated signaling (Supplementary Fig. S5b). The anti-inflammatory activity of IL-10^+^ F-sEVs was further validated using primary human macrophages isolated from peripheral blood mononuclear cells (PBMCs) (Supplementary Fig. S6a). Interestingly, naïve F-sEVs exhibited comparable anti-inflammatory effects to IL-10^+^ F-sEVs in these primary cells, suggesting that endogenous anti-inflammatory mechanisms may be more pronounced in primary versus immortalized cell lines. We next examined the temporal dynamics of IL-10^+^ F-sEV-mediated anti-inflammatory effects. Both naïve and IL-10^+^ F-sEVs sustained suppression of pro-inflammatory markers for up to 72 h in both murine (TNFA) and human macrophages (TNFA, IL6, and IL1B) (Supplementary Fig. S6b and S6c). However, the potential effects of cell death in macrophages following LPS stimulation cannot be excluded in these prolonged cultures.

### 3.6. Anti-inflammatory activity is broadly present across IL-10-loaded EV subpopulations

**Fig. 7.**
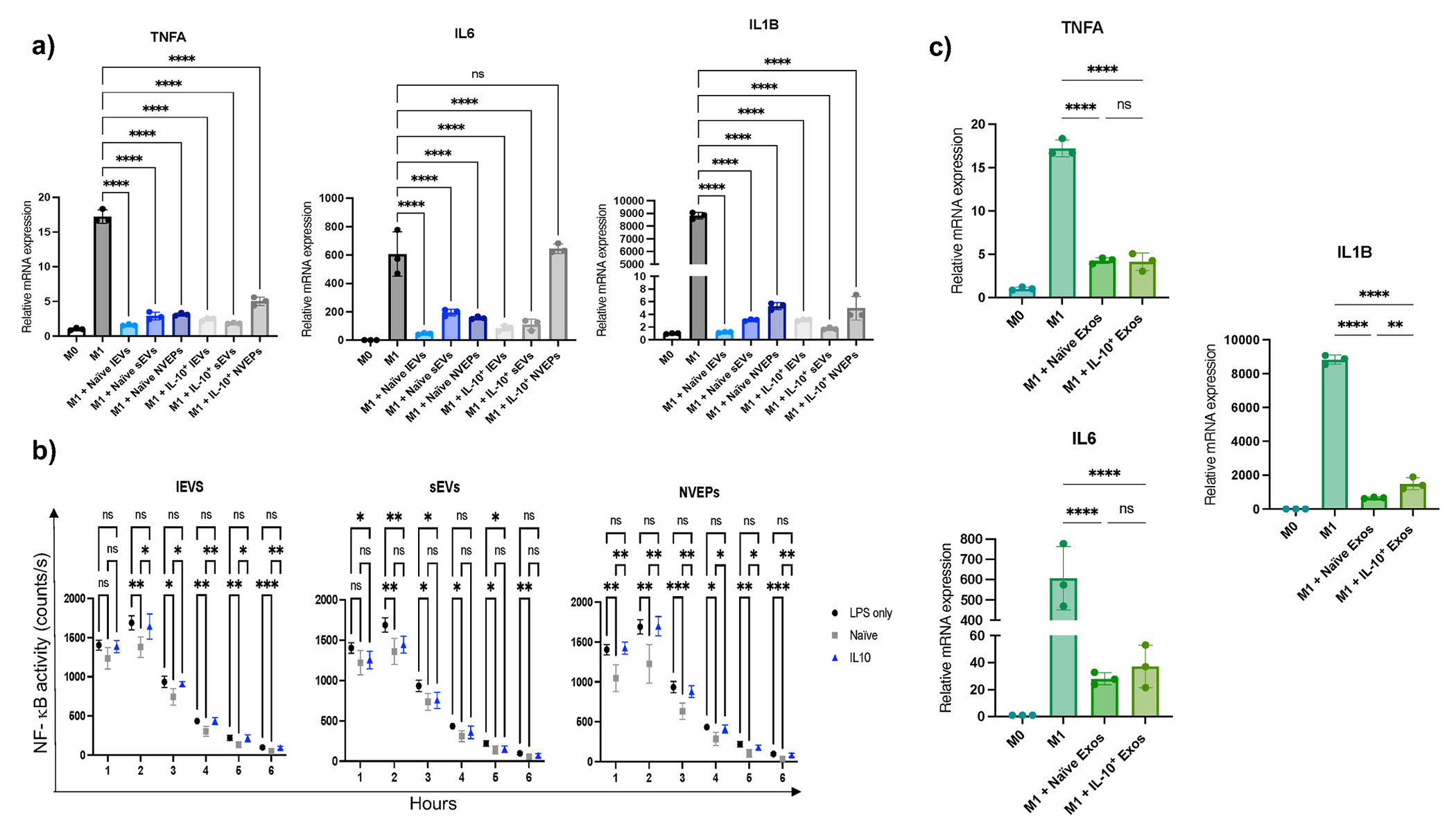
Effect of IL-10^+^ lEVs, sEVs, NVEPs, and exosomes on inflammatory macrophages. **a)** qPCR analysis of pro-inflammatory marker mRNA expression in LPS-stimulated THP-1 macrophages following 24-h incubation with 50 μL of lEVs, UC-sEVs, and NVEPs. (n = 3, one-way ANOVA). **b)** NF-κB activity in M1 macrophages measured by bioluminescence during 1–6 h incubation with lEVs, UC-sEVs, and NVEPs (n = 4, two-way ANOVA). **c)** qPCR analysis of pro-inflammatory marker mRNA expression in LPS-stimulated THP-1 macrophages following 24-h incubation with 10 μg (total protein) of exosomes (n = 3, one-way ANOVA). (*) p < 0.05, (**) p < 0.01, (***) p < 0.001, and (****) p < 0.0001.

Functional assays demonstrated that lEVs, UC-sEVs, and NVEPs reduced pro-inflammatory cytokine expression (TNFA, IL6, IL1B) in M1-like macrophages, indicating that their anti-inflammatory effects are not size-dependent. However, IL-10^+^ NVEPs failed to reduce IL6 expression (Fig. 7a). Notably, among the three fractions, only IL-10^+^ UC-sEVs showed sustained suppression of NF-κB activity for up to 5 h compared to LPS-only controls, whereas all naïve cell-derived groups showed reduced NF-κB activity (Fig. 7b). Furthermore, IL-10^+^ exosomes retained their anti-inflammatory effects, effectively reducing pro-inflammatory cytokine expression in M1-like macrophages (Fig. 7c). Intriguingly, naïve exosome-enriched fractions showed comparable anti-inflammatory activity in our experimental settings, suggesting that endogenous IL-10 or other bioactive cargo molecules contribute to the observed immunomodulatory signaling mechanisms.

### 3.7. Serum-free F-sEVs exhibit intrinsic anti-inflammatory effects

Previous studies have highlighted the potential interference of serum-derived sEVs and non-vesicular components in investigations of intercellular communication.^38^ To exclude potential contributions from serum-derived components to the observed anti-inflammatory activity of naïve F-sEVs, HEK293FT cells were cultured in serum-free conditions for F-sEV production. Western blot analysis revealed CD9 was present only in F-sEVs derived from EV-depleted conditioned media, whereas CD63 was retained in F-sEVs from both serum-free and EV-depleted conditioned media (Supplementary Fig. S7a). This CD63 pattern aligns with previous observations of enhanced CD63 levels under serum-free conditions.^38^ Importantly, no significant differences in suppressing NF-κB activity were observed between these groups (Supplementary Fig. S7b), indicating that the anti-inflammatory properties of F-sEVs are intrinsic and independent of external serum-derived components.

## 4. Discussion

Building upon recent advances in engineered EVs for therapeutic cytokine delivery,^9,39^ our study establishes a robust framework for IL-10 loading, stabilization, and functional delivery via EVs to achieve effective immunomodulation. Unlike electroporation^40^ or chemical conjugation,^41^ which can alter vesicular stability and composition, our overexpression-based approach enables natural incorporation of IL-10 into EVs while preserving native vesicular architecture and cargo integrity. This strategy allowed us to mechanistically dissect both surface-bound and luminal cytokine interactions within physiologically relevant vesicular compositions, addressing a critical gap in understanding EV-mediated cytokine delivery mechanisms. Given their demonstrated anti-inflammatory effects, IL-10^+^ EVs hold significant potential as a therapeutic strategy for inflammatory disorders. Compared to recombinant IL-10 therapy, which suffers from rapid systemic clearance and limited tissue penetration,^8^ IL-10^+^ EVs provide sustained immunomodulatory effects through enhanced stability and efficient deep-tissue delivery, while reducing systemic off-target effects. These advantages highlight the biological benefits of EVs over synthetic carriers such as liposomes, including superior biocompatibility, improved tissue access, and amenability to genetic engineering.

Our analysis revealed two distinct oligomeric forms of IL-10 associated with F-sEVs, in addition to the monomeric form, indicating a unique structural complexity in sEV-associated cytokines, even though monomeric and oligomeric IL-10 showed comparable stability (Fig. 2e). Besides the IL-10 oligomers found in the F-sEV lumen, IL-10 monomers were efficiently retained on the external surface of F-sEVs, suggesting that a corona formation might play a role. Recent studies have revealed that molecules such as DNA and proteins can adsorb onto EV surfaces from the extracellular environment, forming a dynamic EV surface corona.^42^ This corona acts as a dynamic interface for communication between EVs and their surroundings.^43^ However, in our experiment, we could not reproduce this effect by artificially decorating F-sEV surfaces with recombinant IL-10 protein (Supplementary Fig. S3c). This result suggests that the formation of IL-10-containing protein corona may form naturally and spontaneously during biogenesis.^44^ Previous studies have demonstrated that cytokines commonly bind to glycosaminoglycan (GAG) side chains of proteoglycans on EV surfaces,^35^ with these cytokine-GAG interactions exhibiting remarkable specificity even in the absence of cytokine receptors on EVs.^32^ Our heparan sulfate binding assay, however, did not detect IL-10 binding under our experimental conditions (Supplementary Fig. S3b). Nonetheless, we cannot rule out the possibility that IL-10 binds to diverse GAG chains, such as chondroitin sulfate or keratan sulfate. Given the complexity of sEV cargo loading and potential receptor-mediated interactions, further investigation is required to identify the specific molecular mechanisms governing IL-10 association with sEVs.

IL-10^+^ F-sEVs exhibited significant uptake and anti-inflammatory effects in M1-like macrophages, as demonstrated by a marked reduction in key pro-inflammatory cytokines (TNFA, IL6, and IL1B), bringing their levels closer to those observed in M0-like macrophages and sustained reduction of NF-κB activity in pro-inflammatory macrophages. Reducing inflammatory markers in primary macrophages reinforces that the effect is not limited to immortalized cell lines such as THP-1 cells. Unlike previous studies on IL-10,^9^ we did not observe the repolarization of M1-like macrophages to an M2-like phenotype, suggesting that the observed effects were immunosuppressive rather than polarizing. This discrepancy may be due to differences between in vitro and in vivo conditions, as tissue environments facilitate complex interactions among diverse immune cells, which may promote M1 to M2 macrophage repolarization. Notably, surface-exposed IL-10 on F-sEVs selectively downregulated TNFA expression without significantly affecting IL6 or IL1B levels (Supplementary Fig. S6b). This selective effect aligns with previously reported IL-10-mediated anti-inflammatory pathways that reduce NF-κB activity through IL-10 receptor engagement.^45^ However, pretreatment with anti-IL-10Rα antibodies failed to block NF-κB activity (Fig. 5d), suggesting an alternative mechanism. One possibility involves signaling within endosomes following sEV internalization, similar to previously reported endosomal TGFβ signaling mediated by EVs.^34^ Alternatively, recent studies using bioluminescent EVs have shown that EV cargo can be re-released from recipient cells,^46^ suggesting that EV-delivered IL-10 may be re-secreted and subsequently mediate signaling through autocrine or paracrine pathways.

Interestingly, size-based fractionation of EVs and NVEPs by sequential UC combined with filter concentration^25^ revealed that IL-10’s anti-inflammatory effects are not exclusively dependent on their sizes. All fractions contained IL-10 and exhibited similar reductions in inflammatory cytokine expression. However, IL-10^+^ UC-sEVs uniquely showed sustained NF-κB suppression in pro-inflammatory macrophages, suggesting a potential functional advantage specific to UC-sEVs (Fig. 7b). Proteinase K protection assays indicated that IL-10 monomers were located on the external surface of both small and large EVs. In contrast, NVEPs efficiently contained both monomers and oligomers of IL-10, with monomers being protected from proteinase K digestion (Fig. 3d), suggesting structural heterogeneity that may influence cytokine release and mechanisms of cellular interaction. Additionally, our findings demonstrate that the isolation process involving serial UC contributes to the loss of the higher-order oligomeric form of IL-10 (≥102 kDa), revealing their sensitivity to this methodology. Previous studies have shown that the high shear forces generated during ultracentrifugation can potentially disrupt EV structure.^47^ While our size-based and charge-based EV purification approaches allowed for separation of vesicle subpopulations, the detection of IL-10 in NVEP fractions highlights the need for further investigation into the potential immunomodulatory contribution of NVEPs, which may confound or complement EV-mediated effects. Anion exchange chromatography purification of IL-10^+^ sEVs, as recently demonstrated,^27^ effectively isolated IL-10-enriched exosomes with potent anti-inflammatory functionality based on their surface charge and high protein content. Notably, exosome fractions derived from naïve and IL-10-transfected HEK293FT cells contained lower molecular weight oligomeric IL-10, whereas monomeric and higher-order oligomers were detected only in IL-10^+^ exosome fractions (Fig. 4c). Despite these molecular differences, both exosome fractions exhibited comparable anti-inflammatory activities in pro-inflammatory macrophages. Although IL-10^+^ EVs demonstrate promising immunomodulatory effects in vitro, several challenges remain for clinical translation.^48^ The structural and compositional complexity of IL-10 associated with EVs and NVEPs poses significant obstacles to precise dosing and reproducible evaluation of therapeutic efficacy. Moreover, batch-to-batch variability in EV preparations, driven by differences in isolation methods, culture conditions, and donor sources, underscores the need for standardized, scalable manufacturing workflows.

Finally, to assess the influence of culture conditions and potential serum contamination on the anti-inflammatory effects of naïve F-sEVs, we isolated F-sEVs under serum-free conditions, as recently demonstrated.^38^ F-sEVs isolated from serum-free medium expressed CD63 but not CD9, and their modulation of NF-κB activity in THP-1 cells occurred independently of serum-derived components. The intrinsic immunomodulatory properties of naïve F-sEVs observed in this study further highlight the complexity of their therapeutic potential. Their anti-inflammatory activity may arise from endogenous bioactive cargo, such as miRNAs,^49^ or from intrinsic structural features of the vesicles, including anti-inflammatory membrane lipids.^50^ Our findings reinforce the emerging view that EVs are not inert carriers, but complex biological entities with inherent immunomodulatory properties shaped by their cellular origin and molecular composition, both surface-associated and internal. This highlights that the biological activity of EV cargo, such as cytokines, cannot be fully understood in isolation, as the contextual presentation of the vesicle itself can reprogram or even dominate functional outcomes. These insights underscore the need for further investigation into the endogenous molecular composition of naïve EVs, which could be strategically leveraged to complement or enhance the efficacy of engineered vesicles.

## Supplementary Material

1

2

## Appendix A. Supplementary data

Supplementary data to this article can be found online at https://doi.org/10.1016/j.vesic.2025.100102.
