# Supplementary material for: Harnessing extracellular vesicles for stabilized and functional IL-10 delivery in macrophage immunomodulation
Source: Extracell Vesicle. Author manuscript; Available in PMC 2026 Jun 26. (PMC13297991; doi:10.1016/j.vesic.2025.100102)
Supplement: 1 [file NIHMS2187194-supplement-1.docx]

**SUPPLEMENTARY INFORMATION**

**Harnessing Extracellular Vesicles for Stabilized and Functional IL-10 Delivery in Macrophage Immunomodulation**

Najla A. Saleh, Matthew A. Gagea, Xheneta Vitija, Sadhana Kilangodi, Admed A. Zarea,

Tomas Janovic, Jens C. Schmidt, Cheri X. Deng, Masamitsu Kanada^*^

^*^Author to whom correspondence should be addressed.

Email: kanadama@msu.edu (M.K.)

**Contents:**

- Supplementary Table 1
- Supplementary Figures S1-S7

| Gene | Forward |  | Reverse |
| --- | --- | --- | --- |
| hIL10 | TACGGCGCTGTCATCGATTT |  | TAGAGTCGCCACCCTGATGT |
| hIL6 | ACTCACCTCTTCAGAACGAATTG |  | CCATCTTTGGAAGGTTCAGGTTG |
| hIL1B | ATGATGGCTTATTACAGTGGCAA |  | GTCGGAGATTCGTAGCTGGA |
| hTNFA | CCTCTCTCTAATCAGCCCTCTG |  | GAGGACCTGGGAGTAGATGAG |
| hMRC1 | TGGTTTCCATTGAAAGTGCTGC |  | TTCCTGGGCTTGACTGACTGTTA |
| hCD209 | AAATCAGGAAGGCACGTGGCAT |  | GTTGGGCTCTCCTCTGTTCCAA |
| hCD163 | GGATGTCCAACTGCTATCAA |  | GACTCATTCCCACGACAAGAA |
| hCD68 | GCTACATGGCGGTGGAGTACAA |  | ATGATGAGAGGCAGCAAGATGG |
| hGAPDH | GGGTGTGAACCATGAGAAGT |  | GGCATGGACTGTGGTCATGA |
| mTNFA | ATGAGCACAGAAAGCATGATC |  | TACAGGCTTGTCACTCGAATT |
| mGAPDH | TGCTGAGTATGTCGTGGAGT |  | GTTCACACCCATCACAAACA |

**Supplementary Table 1. List of primers used in this study**. h: Human; m: Mouse.

**Supplementary Figure S1. Enhanced and sustained IL-10 overexpression via MC-IL10 transfection**. **a)** Agarose gel electrophoresis confirms the ability to generate parental plasmid (PP)- and MC-IL10. DNAs were digested with HindIII. **b)** HEK293FT cells transfected with MC-IL10-RFP. Fluorescence signals of RFP (red; mScarlet) were merged with nuclei stained with Hoechst 33342 (blue). **c)** IL10 expression (cell lysate) was confirmed by Western blotting and qPCR, while IL10-protein and -mRNA were not detected in HEK293FT cells without transfection (TF). GAPDH indicates a loading control. (n=3, unpaired two-tailed Student’s t-test). **d)** Fluorescence microscopy analysis of IL10-RFP expression in HEK293FT cells transfected with equal masses (100 ng/well) of PP vectors (size: 5.7 kbp) or MC vectors (size: 1.7 kbp) in a 96-well plate. Images were captured under the same acquisition parameters (exposure time, gain, and illumination intensity) to enable quantitative comparison of fluorescence signals. **e)** Time-course analysis of IL10-RFP expression from PP and MC vectors measured by fluorescence plate reader over time (n = 5). Fluorescence values were obtained by subtracting the autofluorescence signals from the untransfected control wells.

**Supplementary Figure S2. IL-10 association with F-sEV subpopulations. a)** Transmission electron microscopy of HEK293FT cell-derived IL-10^+^ PalmReNL-F-sEVs immunogold labeled for IL-10 (yellow arrow) and small particles (< 50 nm - orange arrows). Scale bar = 100 nm.  **b)** Western blot analysis of TSG101 EV marker and Calnexin, an endoplasmic reticulum protein used as an EV negative marker (Cell lysate was used as positive control for anti-Calnexin antibodies) under reducing conditions. An equal amount (30 μg) of total protein of F-sEVs was used for Western blot analysis. **c)** Single-EV analysis using fluorescent microscopy to characterize the co-localization (yellow arrows) of IL-10-RFP^+^ F-sEVs (red) with EV marker tetraspanins—CD9-GFP, CD63-GFP, or CD81-GFP (green). Scale bar = 1 μm.

**Supplementary Figure S3. Characterization of IL-10 binding to F-sEVs. a-c)** Western blot analysis of IL-10 protein expression in: **(a)** HEK293FT cells following treatment with the inhibitor of heparan sulfate proteoglycan (HSPG) biosynthesis, 4-nitrophenyl β-D-xylopyranoside (PNP-Xyl); **(b)** IL-10^+^ F-sEVs after heparinase treatment (H+); and **(c)** naïve F-sEVs incubated with recombinant human IL-10 protein (hIL10 RP) at room temperature (RT). An equal amount (30 μg) of total sEV protein was used for each condition.

**Supplementary Figure S4. Characterization of Protein-high IL-10^+^ exosome fractions after purification.** Size distribution histogram showing particle concentration per size interval (particles/mL). The particle concentration, peak size, total protein concentration, and particle/protein ratio of fractions 9, 10, 11, and 12 were included in the figure.

**Supplementary Figure S5. Effects of recombinant IL-10 protein and IL-10^+^ F-sEVs on pro-inflammatory macrophages.** qPCR analysis of **(a)** anti-inflammatory and **(b)** pro-inflammatory marker mRNA expression in LPS-stimulated THP-1 macrophages following 24 h incubation with F-sEVs (10 µg total protein) or recombinant IL-10 protein (IL-10 RP; 10 ng/mL). PK: proteinase K-treated F-sEVs. (n=3, one-way ANOVA). (*) p < 0.05, (**) p < 0.01, (***) p < 0.001, and (****) p < 0.0001.

**Supplementary Figure S6. Effects of IL-10^+^ F-sEVs on pro-inflammatory macrophages from various sources.** qPCR analysis of pro-inflammatory marker mRNA expression in LPS-stimulated **(a)** human primary macrophages from peripheral blood mononuclear cells (PBMCs), **(b)** RAW 264.7, and **(c)** THP-1 macrophages following incubation with F-sEVs (10 µg total protein) for 24 h, 48 h, and 72 h (n=3, one-way ANOVA). (*) p < 0.05, (**) p < 0.01, and (****) p < 0.0001.

**Supplementary Figure S7. Characterization of F-sEVs isolated from serum-free versus EV-depleted serum media. a)** Western blot analysis of CD9 and CD63 in F-sEVs isolated from serum-free (SF) and EV-depleted (Reg) conditioned media, non-reducing condition **b)** NF-κB activity in monocytes measured by bioluminescence assay following incubation with SF or Reg F-sEVs at low (2 µg) or high (20 µg) protein concentrations (n=4, one-way ANOVA). (****) p < 0.0001.
