# Supplementary material for: Harnessing extracellular vesicles for stabilized and functional IL-10 delivery in macrophage immunomodulation
Source: Extracell Vesicle. Author manuscript; Available in PMC 2026 Jun 26. (PMC13297991; doi:10.1016/j.vesic.2025.100102)

TSG101 - Cy5 channel. At the beginning of our experiments, a dot was made, at 17 (blue) and 102 (green) kDa, in the membrane.

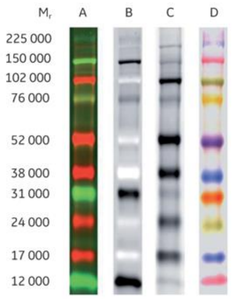

Fig 3. Amersham ECL Plex Fluorescent Rainbow Molecular Markers imaged on a Typhoon scanner. From left to right: (A) Full-color Cy3 and Cy5; (B) Cy3 channel; (C) Cy5 channel; and (D) visible spectrum.

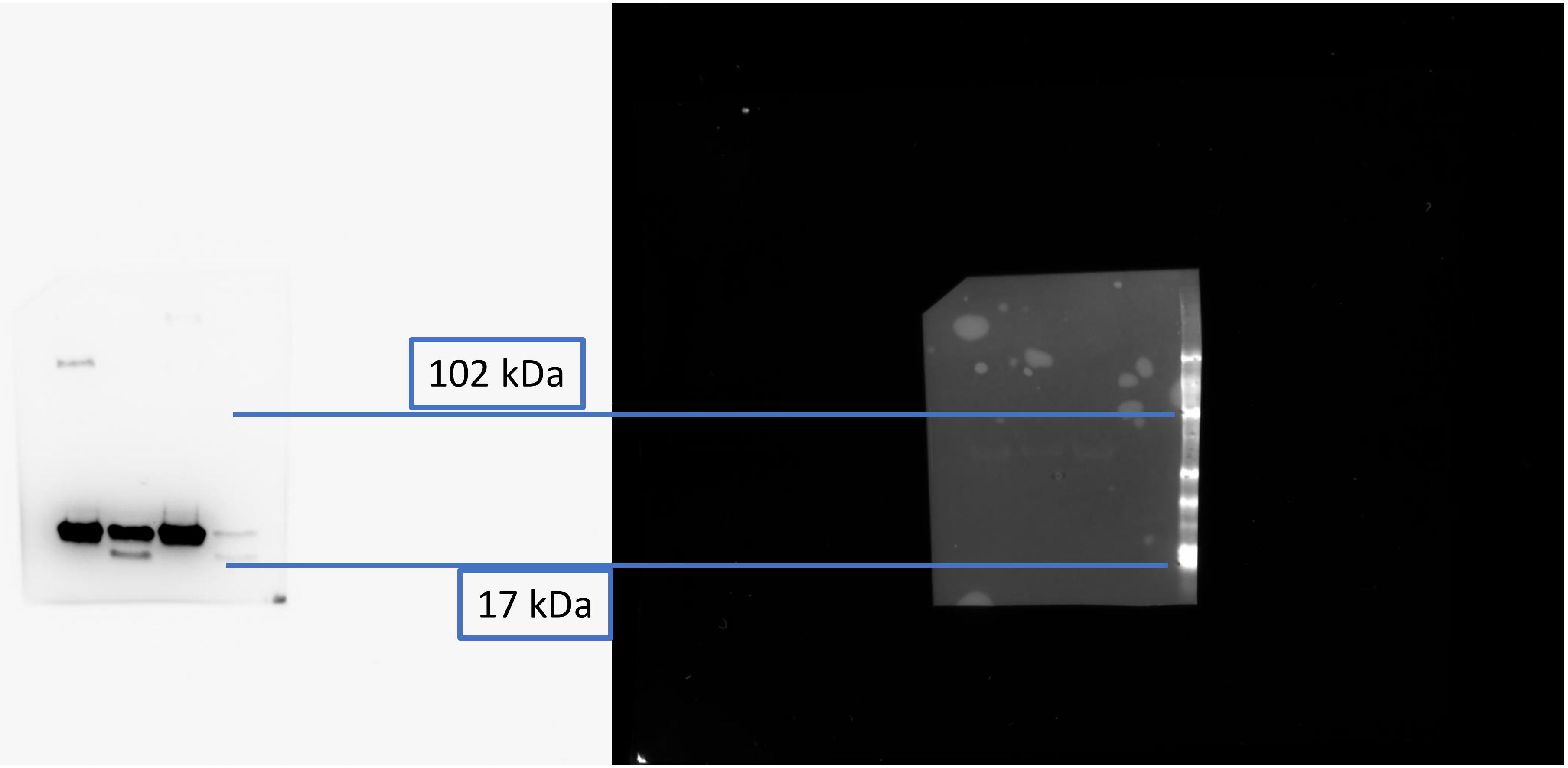

CD91

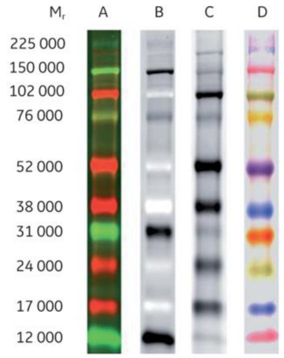

Fig 3. Amersham ECL Plex Fluorescent Rainbow Molecular Markers imaged on a Typhoon scanner. From left to right: (A) Full-color Cy3 and Cy5; (B) Cy3 channel; (C) Cy5 channel; and (D) visible spectrum.

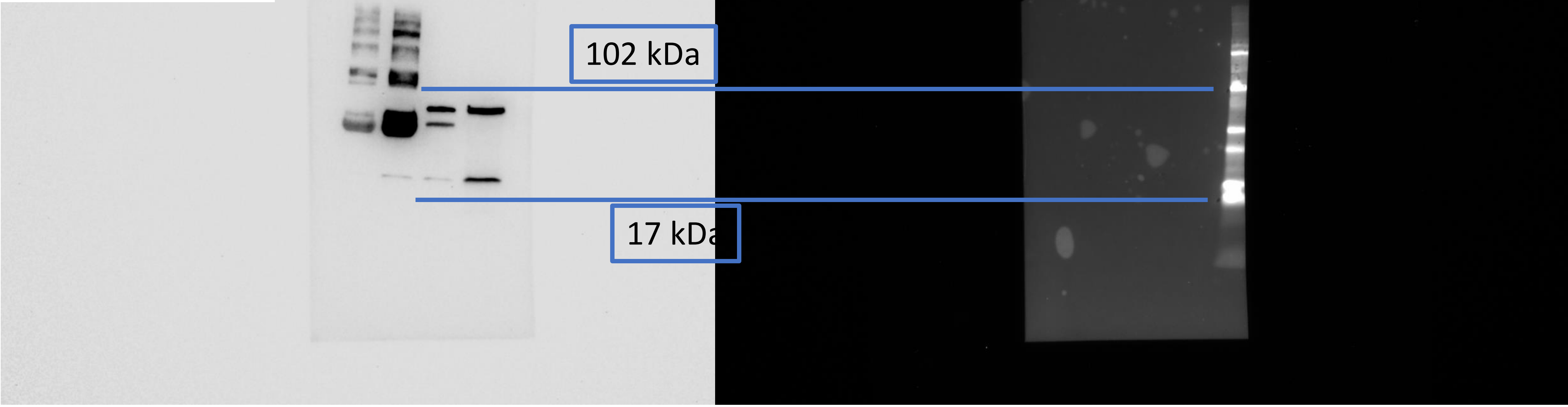

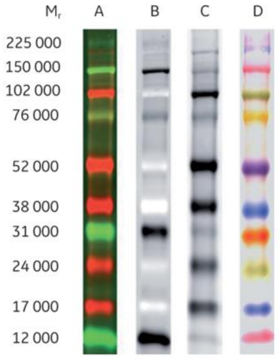

Fig 3. Amersham ECL Plex Fluorescent Rainbow Molecular Markers imaged on a Typhoon scanner. From left to right: (A) Full-color Cy3 and Cy5; (B) Cy3 channel; (C) Cy5 channel; and (D) visible spectrum.

CD81

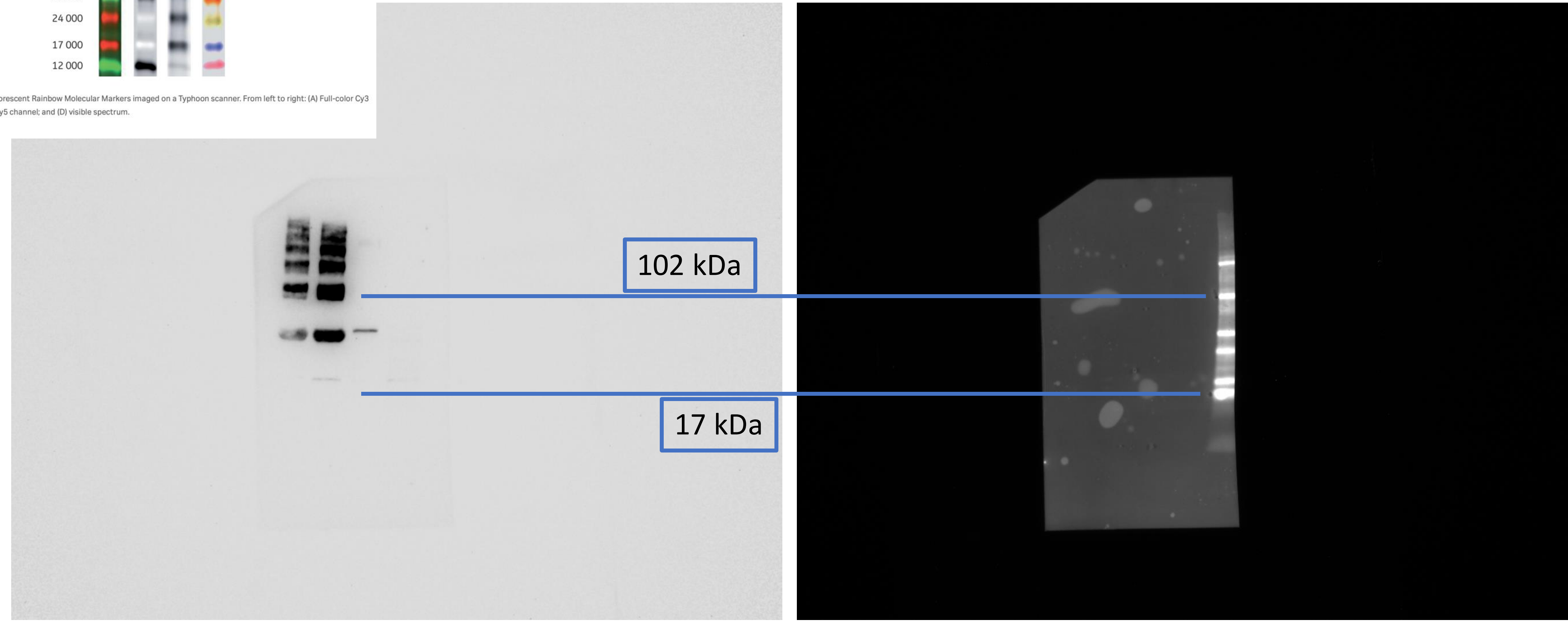

IL10 - Cy5 channel. Later, we started to make the dots on all Cy5 bands: 17 (blue), 24 (green), 38 (blue), 52 (purple), 102 (green) kDa.

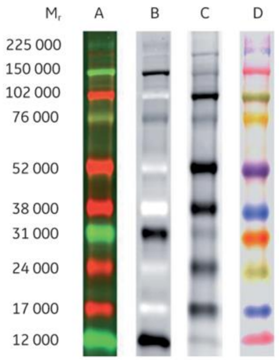

Fig 3. Amersham ECL Plex Fluorescent Rainbow Molecular Markers imaged on a Typhoon scanner. From left to right: (A) Full-color Cy3 and Cy5; (B) Cy3 channel; (C) Cy5 channel; and (D) visible spectrum.

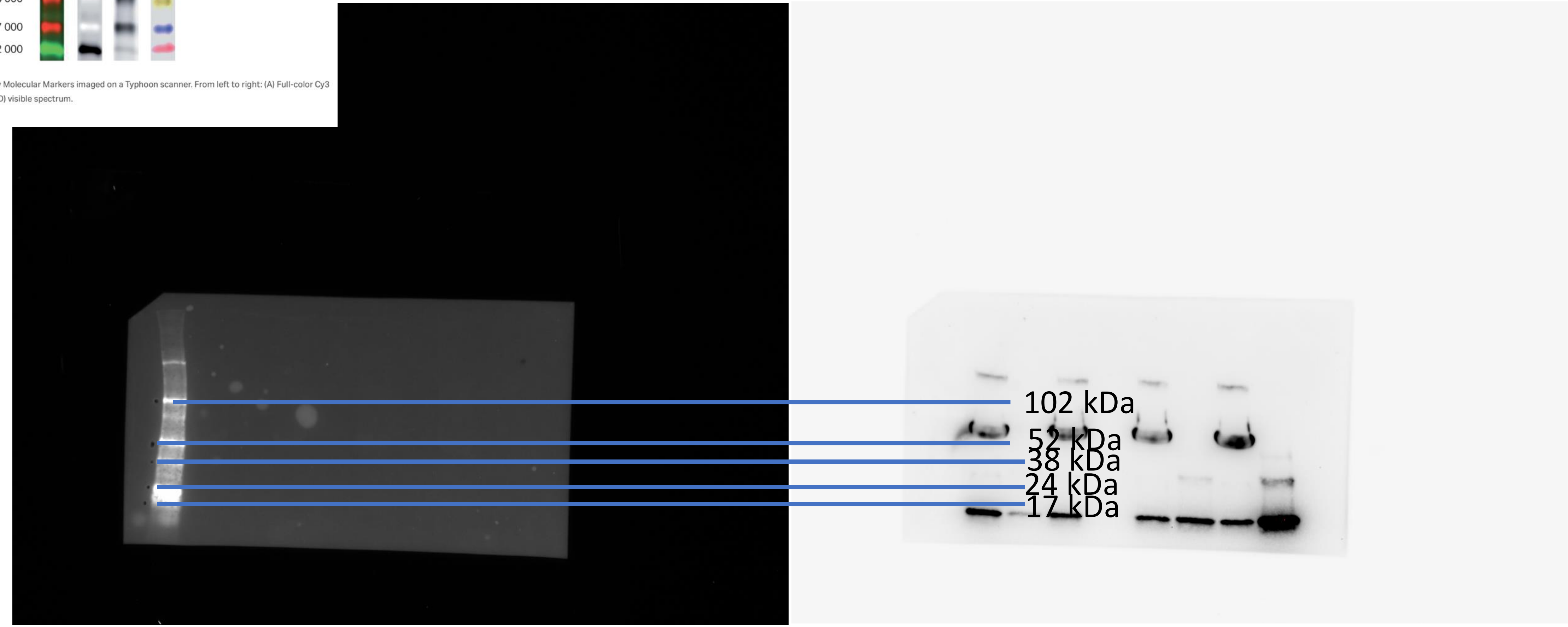

Supplement: 2 [file NIHMS2187194-supplement-2.pdf]
